# Quality of life associated with oral health among patients with single versus two-implant retained mandibular overdentures

**DOI:** 10.6026/973206300213288

**Published:** 2025-09-30

**Authors:** Chaithanya Ramisetty Nagabhushan, Srinidhi Bhat, Ananya R.M., Dimple M., Jaya Krishna T., Swathi K. Bhat, Naveen Sai Ramisetty Nagabhushan

**Affiliations:** 1 Department of Prosthodontics, Sharavathi Dental College & Hospital, Shimoga, Karnataka, India; 2 Department of Prosthodontics, Srinivas Institute of Dental Sciences, Mangalore, Karnataka, India; 3 Department of Prosthodontics, Oxford Dental College, Bangalore, Karnataka, India; 4 Department of Dentistry, Vokkaligara Sangha Dental College & Hospital, Bangalore, Karnataka, India

**Keywords:** Oral health related quality of life, implant retained overdentures, early loading, elderly, edentulous mandibular arches

## Abstract

Evaluation of Oral Health-Related Quality of Life (OHRQoL) among patients with single versus two-implant retained mandibular over dentures is of interest. Therefore, a prospective research was doneon 16 patients with Group A (Single implant retained overdentures group) and Group B (Two implant retained overdenture group). Subjective parameters were assessed using OHIP-14 questionnaire. Thus, single implant retained overdenture can be considered as a promising treatment modality for mandibular edentulous arches.

## Background:

Medical advancements as well as other social and economic variables are contributing to an increase in life expectancy, leading to an increase in elderly edentulous population and increase in demand for oral health care services [1]. For the past few decades, conventional complete dentures (CCD) were considered as the primary choice of treatment for edentulous patients as there were not numerous treatment options. These CCD satisfied most of the essential needs of the patients such as masticatory capacities, aesthetics etc. In any case, this removable prosthesis has its drawbacks, particularly the mandibular dentures such as need of maintenance, retention and stability [2]. According to McGill consensus and York consensus at the annual conference of BSSPD (British Society for the study of Prosthetic Dentistry) in York, UK 2009 suggested that the conventional complete dentures are no longer considered as the first choice of treatment for edentulous patients instead two implant supported over dentures are considered to be the first choice of treatment [3]. Several studies have shown there is an improved patient satisfaction, chewing efficiency, bite force and requires less maintenance [4, 5]. A direct cost comparison study was conducted, which revealed that two implant supported overdenture costs 2.4 times more than that of CCD whereas the single implant supported overdenture costs around 1.6 times more than that of the conventional complete dentures. Currently the single implant retained overdenture has shown success in the treatment outcome and is also cost effective [6]. Therefore, it is of interest to assess the oral health related quality of life between single and two implant retained overdentures.

## Materials and Methods:

This prospective observational comparative study was conducted to evaluate the life quality between single and two implant retained overdentures. This study included a total of 16 patients (7 female, 9 male) and were divided into two groups. Each patient was assigned a number according to the date of enrollment in the study. Group A: In 8 patients single implant is placed in the midline of the mandibular edentulous ridge. Group B: In 8 patients two implants are placed, one on each side in the canine region of the mandibular edentulous ridge. The inclusion criteria included patients with age ranging from ≥30 to ≤80 years, completely edentulous patients, sufficient volume of bone in anterior region of the mandible, patients who were unsatisfied with their previous dentures. The exclusion criteria were patients with any local and systemic diseases, chronic smokers and tobacco chewers, patients undergoing radiation therapy or chemotherapy, patients under prophylactic antibiotics or steroids. The implants (OSSTEM) were placed 0.5-1mm below the level of the crestal bone in the midline and in the canine regions respectively and is considered as 0th day (Baseline). At the end of 60 days (Two months). Overdenture abutment/s (OSSTEM, Stud abutments) were torqued onto the respective implant. Maxillary and mandibular dentures were fabricated in conventional manner preoperatively. In the mandibular denture, tissue surface corresponding to the implant site will be trimmed. The female component will be positioned over the overdenture abutment. The metal housing (female component) with the retentive component were picked up in the denture from the overdenture abutment with self-cure acrylic resin. At 5th month the OHIP-14 scores were recorded. The Data obtained from the study was compiled on MS Office Excel Sheet (v 2019, Microsoft Redmond Campus, Redmond, Washington, United States). The Statistical Package for Social Sciences (SPSS v 26.0, IBM) software was used to statistically analyse the data with P<0.05. Parametric tests, such as Independent sample t-tests (Unpaired-t test) were used for subsequent comparative analyses.

## Results:

In this prospective clinical study a total of 16 patients were evaluated, with 8 in each group. All the implants survived with 100 percent success rate. After 3 months of loading (5th month) the OHIP scores were recorded and compared. Inter group comparison of OHIP scores between group A (7.50± 1.41) and group B (7.62± 1.41) was done by using an independent sample t-test. The results revealed that there were no statistically considerable variation among the groups at defined time interval (p >0.05) (Table 1 - see PDF).

## Discussion:

This non-randomized prospective clinical study was planned, to examine the difference between the single and two implant overdentures in terms of oral health related quality of life. This study was performed using the best available methodology according to principles of evidence based medicine. "During the 5 month follow up period the overall success rate was good and there was no failure in both the groups when the implants were early loaded", indicating single implant overdenture could be a substitute to two implant overdenture as it is less technique sensitive and less invasive. The single implant overdenture can be considered as a promising treatment modality in elderly edentulous population especially in population with lower socioeconomic status. This discovery was in agreement of a previous study by Kronstrom *et al.* in 2017, where the patient satisfaction and clinical outcomes were assessed using OHIP-14 questionnaire concluded that there was very minimal need for the maintenance of the prosthesis and the patient's satisfaction was higher even after the implants were loaded immediately in both single and two implant overdenture groups [4]. In this clinical study the OHIP scores were slightly higher in the two implant retained overdenture group (7.62± 1.41) when compared to the single implant retained overdenture group (7.50± 1.41). However, the difference in the OHIP scores were not statistically significant (p>0.05). This indicates that both single implant and two implants has almost similar outcomes at 5 months follow up period with regards to oral health related quality of life when used for implant retained overdentures in an edentulous mandible. In A review by Mahoorkar *et al.* Cost comparison studies have demonstrated that single-implant retained mandibular overdentures (SIROD) are notably more affordable than two-implant supported overdentures (TISOD) [6]. In a study conducted by Cordioli *et al.* in 1997, where single implants were placed in the mandibular midline using the standard two-stage surgical protocol. The findings demonstrated that single implants at the mandibular midline can provide effective and reliable support for overdentures over the medium term. Throughout the observation period, no implant failures were recorded, and the success rate was found to be comparable to that of overdentures supported by multiple implants. These results support the viability of single-implant mandibular overdentures as a cost-effective and stable alternative, especially for patients where multiple implants may not be feasible [7].

According to Walton and MacEntee, TISODs are approximately 1.75 times more expensive than SIRODs [8]. Another study by Takanashi *et al.* reported that two implant supported overdentures costs2.4 times more than complete dentures, whereas single implant overdentures cost 1.31 times that of the traditional complete dentures. The reduced cost of SIROD is attributed to simpler surgical procedures, fewer implants, and minimal maintenance requirements. This makes SIROD an attractive option for financially constrained edentulous patients, particularly in developing regions. Importantly, despite the lower cost, SIROD demonstrates comparable success in terms of clinical performance and patient satisfaction when compared to TISOD, making it a practical and economical treatment choice [9]. This aligns with the conclusions of the McGill (2002) and York (2009) consensus statements, which recommend a minimum of two-implant supported overdentures (TISOD) as the standard treatment for the edentulous mandible. Multiple randomized trials and systematic reviews show that ISODs greatly enhance patient satisfaction, oral health-related quality of life, chewing function, and social confidence when compared with conventional dentures. Additionally, ISODs help slow further bone resorption, boast approximately 95% long-term survival rates, and prove cost-effective over time-even when accounting for higher initial investment-due to fewer complications, improved upkeep, and better quality of life. This body of evidence supports their broader adoption and indicates the need for more training so that implant-retained overdentures can be routinely offered as the first choice for edentulous mandibular patients [3]. A clinical crossover study conducted by Bhat *et al.* in 2016evaluated for chewing efficiency and overall satisfaction using four different setups: conventional dentures and overdentures anchored by one, two, or three implants. This study assessed the performance of mandibular overdentures supported by one, two, and three implants within the same group of edentulous patients. The observations suggested that even a single implant in the midline of edentulous mandibular ridge significantly improved the bite force when compared to that of traditional complete dentures. Although satisfaction did not notably increase with just one implant, it improved considerably with the addition of two and three implants, with the highest results seen in the three-implant configuration. The study suggests that single implant overdentures offer a practical and affordable improvement over conventional dentures [10]. Geertman *et al.* came to the conclusion that the transmandibular implant group had much better surgical outcomes than the endosseous implant group [11]. According to Husain *et al.*'s evaluation of the disparity in oral health-related quality of life, urban areas have higher oral health-related quality of life than rural ones [12]. Paredes-Rodríguez *et al.* claim that neither the quantity of drugs consumed nor the number of left teeth are associated with quality of left life are associated On the other hand, a lower quality of life was substantially linked to a higher degree of xerostomia [13]. Implant failure occurs when peri-implantitis is left untreated. Identification of the peri-implant state is aided by peri-implant crevicular fluid (PICF) [14]. According to Patil *et al.* older edentulous people's quality of life is improved by mandibular single and implant-retained overdentures [15]. In a research by Patil *et al.* patient satisfaction rose by 11.4% in a month and by 16.2% in a year for patients with a single implant, and by 14.3% in a month and 18.4% in a year for patients with two implants [16].

## Conclusion:

The OHIP scores were slightly higher in two implant retained overdenture group when compared to the single implant overdenture group. However the results were statistically insignificant. Hence, single implant retained overdenture can be considered as a promising treatment modality in elderly patients.

## References

[1] Thomason JM (2009). Br Dent J..

[2] Grover M (2014). Clin Oral Implants Res..

[3] Thomason JM (2012). J Dent..

[4] Kronstrom M (2017). Int J Oral Maxillofac Implants..

[5] Boven GC (2015). J Oral Rehabil..

[6] Mahoorkar S (2016). J Indian Prosthodont Soc..

[7] Cordioli G (1997). J Prosthet Dent..

[8] Bryant SR (2015). J Dent Res..

[9] Takanashi Y (2004). Int J Prosthodont..

[10] Bhat S (2016). J Indian Prosthodont Soc..

[11] Geertman ME (1996). Int J Oral Maxillofac Implants..

[12] Husain FA (2017). Open Dent J..

[13] Paredes-Rodríguez VM (2016). J Clin Exp Dent..

[14] Bahadur S (2025). Bioinformation..

[15] Patil PG (2021). J Indian Prosthodont Soc..

[16] Patil PG (2018). J Indian Prosthodont Soc..

